# Soccer Players’ Physical Performance After Loaded Plyometric-Jump Training: A Systematic Review

**DOI:** 10.3390/jfmk11030280

**Published:** 2026-07-20

**Authors:** Miguel Alarcón-Rivera, Joaquín Salazar-Méndez, Eduardo Guzmán-Muñoz, Rodrigo Ramirez-Campillo, Ivan Castillo Montecinos, Luis Arturo Gómez-Landero

**Affiliations:** 1 School of Sports Sciences and Physical Activity, Faculty of Health, Universidad Santo Tomás, Talca 3460000, Chile; jsalazar13@santotomas.cl; 2 Escuela de Doctorado, Universidad Pablo de Olavide, 41013 Sevilla, Spain; 3 School of kinesiology, Faculty of Health, Universidad Santo Tomás, Talca 3460000, Chile; eguzmanm@santotomas.cl; 4 School of Physical Education, Faculty of Education, Universidad Autónoma de Chile, Talca 3460000, Chile; 5 Exercise and Rehabilitation Sciences Institute, Faculty of Rehabilitation Sciences, Universidad Andres Bello, Santiago 7591538, Chile; rodrigo.ramirez@unab.cl; 6 Department of Physical Activity Sciences, Universidad de Los Lagos, Osorno 5290000, Chile; 7 Faculty of Medicine, Universidad Católica del Maule, Talca 3480112, Chile; 8 Physical Performance & Sports Research Center, Universidad Pablo de Olavide, 41013 Sevilla, Spain; lagomrod@upo.es

**Keywords:** additional weight, athletic performance, explosive strength, football, muscle strength, neuromuscular adaptations, resistance training, stretch-shortening cycle, training adaptation

## Abstract

**Objective:** This systematic review examined the effects of loaded plyometric-jump training (LPJT) on physical performance outcomes in soccer players. **Methods:** A systematic search of PubMed, Web of Science, Scopus and Embase covered all records from database inception to February 2026, with an additional update performed in March 2026. Eligible studies were randomized controlled trials comparing LPJT with unloaded plyometric-jump training (UPJT) or passive control conditions. Methodological quality was assessed using the PEDro scale, risk of bias using RoB 2, and certainty of evidence using the GRADE approach. Findings were synthesized narratively. **Results:** Six randomized controlled trials involving 214 soccer players met the inclusion criteria. In general, LPJT showed favorable within-group changes in selected physical performance outcomes, particularly horizontal jump performance and some strength- and power-related variables. However, comparable improvements were also observed after UPJT in several outcomes, including vertical jump performance, linear sprint performance, change-of-direction and agility performance, and soccer-specific outcomes. Five studies raised some concerns, and one study presented a high overall risk of bias. The certainty of evidence ranged from low to very low across outcomes. **Conclusions:** Current evidence remains highly uncertain and does not allow firm conclusions regarding the superiority of LPJT over UPJT or passive control conditions. LPJT may therefore be considered a possible alternative to UPJT rather than a demonstrably superior approach. LPJT may be associated with favorable changes in selected performance outcomes. However, these findings should be interpreted cautiously because of the small evidence base, limited sample sizes, methodological heterogeneity, residual risk of bias concerns, and low to very low certainty of evidence. Further well-designed randomized controlled trials are needed to clarify whether LPJT provides additional benefits beyond conventional unloaded plyometric training.

## 1. Introduction

Soccer is an acyclic, intermittent sport characterized by repeated high-intensity actions interspersed with lower-intensity recovery periods [1]. Athletes must develop a broad spectrum of physical performance skills, including jumping, accelerating, sprinting and decelerating, among others [2]. Physical training constitutes an essential element in soccer, as it consistently contributes to the improvement of physical performance parameters and the prevention of injuries [3]. Specifically, resistance training prepares players to meet the growing physical demands associated with contemporary soccer competition [4,5]. Among the various resistance training methods, plyometric training (PT) has emerged as a particularly effective approach for enhancing physical performance and developing specific skills, such as ball striking in soccer [6,7,8]. PT can enhance lower limb muscle strength, power, sprinting and bone mineral density through neuromuscular adaptations integral to the muscle contractions involved in PT [9].

This method includes jumps with body weight (BW) or external loads in various movement planes [10]. In PT, the muscle stretch-shortening cycle plays a fundamental role, being involved in both its fast and slow phases [11]. The efficacy of the stretch-shortening cycle is associated with various mechanisms, including elastic energy accumulation [12], preload [13], stretch reflexes and muscle–tendon interactions [14]. These contribute to augmented mechanical work output during subsequent concentric muscle contractions [14]. Recently, loaded plyometric-jump training (LPJT) has gained increasing attention as a promising variation of traditional plyometric training aimed at improving physical attributes relevant to soccer performance, including muscle strength, sprint ability, and jump performance [15,16,17]. LPJT differs from unloaded plyometric training (UPJT) primarily by the addition of external load, commonly prescribed relative to BW, with approximately 8% BW frequently reported and typically applied using handheld weights or weighted vests [16,17]. These adaptations may be particularly relevant for soccer players, as they underpin explosive sprinting, jumping performance, and rapid changes of direction, while potentially contributing to injury prevention in high-demand competitive environments [18].

Previous reviews have examined plyometric-jump training programming in soccer players [7], plyometric training effects on soccer-specific outcomes such as kicking performance [8], and the broader optimization of plyometric-jump training exercises across mixed populations [19]. However, these reviews have not specifically synthesized randomized controlled trials examining loaded plyometric-jump training in soccer players or clarified whether LPJT provides additional benefits compared with unloaded plyometric-jump training or passive control conditions.

Therefore, this systematic review aimed to critically examine the effects of LPJT on soccer players’ physical performance, compare these effects with those of UPJT or passive control conditions, and assess the methodological quality and certainty of the current body of evidence.

## 2. Materials and Methods

### 2.1. Protocol and Register

This systematic review was conducted and reported in accordance with the PRISMA 2020 (Preferred Reporting Items for Systematic Reviews and Meta-analyses) recommendations [20]. The study protocol was prospectively registered in the Open Science Framework (OSF) database on 6 February 2025, under the identifier at https://doi.org/10.17605/OSF.IO/MZ6DR.

### 2.2. Eligibility Criteria

The study selection criteria were established according to the PICOS model (Population, Intervention, Comparison, Outcome, and Study design) principles, as outlined by Considine et al. [21]. The eligibility criteria were defined as follows:

(1) Population: soccer players of any age, sex, and competitive level.
(2) Intervention: LPJT, defined as jump training performed with an external load, such as dumbbells, weighted vests, or specific loading devices, aimed at improving the ability to effectively use the stretch-shortening cycle and the rapid transition from eccentric to concentric muscle actions [22,23]. Studies were eligible when LPJT was applied as the main experimental intervention and was not combined with other structured training methods within the same experimental session. Specifically, studies in which the LPJT group performed additional unloaded plyometric jump training, sprint training, change-of-direction (COD) drills, resistance training, or other complementary training modalities as part of the intervention were excluded to isolate the effects of the loaded plyometric stimulus. However, studies in which participants continued their regular soccer-specific training were not excluded, provided that this training was comparable between groups.
(3) Comparison: UPJT or passive control conditions.
(4) Outcomes: physical performance outcomes, including jump performance, muscular strength, linear sprint performance, agility or COD ability, and cardiovascular endurance.
(5) Study design: randomized controlled trials published in peer-reviewed journals, written in any language, from inception to 31 March 2026.

The exclusion criteria were: (1) studies in which LPJT was combined with other structured training interventions as part of the experimental protocol; (2) pre-experimental studies without a control group; and (3) editorials, letters, narrative reviews, systematic reviews, meta-analyses, case reports, and conference abstracts.

### 2.3. Information Sources

A comprehensive literature search was conducted in four electronic databases: PubMed/MEDLINE, Scopus, Web of Science, and Embase. The search covered all records from database inception to February 2026, with an additional update performed in March 2026. No restrictions were applied regarding language or publication year.

The search strategy was developed using free-text terms related to the population and intervention and was adapted to the syntax of each database. Search terms included concepts associated with soccer players, football players, plyometric training, loaded plyometric jump training, external load, additional load, weighted jump, resisted jump, weighted vest, vertical jump, and the stretch-shortening cycle. The general search structure was as follows: (“plyometric training” OR “plyometric jump training” OR “loaded plyometric” OR “loaded plyometric jump training” OR “loaded jump” OR “weighted jump” OR “resisted jump” OR “weighted vest” OR “external load” OR “additional load” OR “vertical jump” OR “stretch-shortening cycle”) AND (“soccer players” OR “football players”). The PubMed/MEDLINE strategy did not include MeSH terms and relied on free-text terms, as reported in Supplementary Table S1. The complete database-specific strategies and the number of records retrieved are presented in Supplementary Table S1. The reference lists of the included studies were manually screened. No formal forward citation searching or grey literature search was performed.

### 2.4. Study Selection Process and Data Collection

All records retrieved from the database searches were imported into Rayyan software (https://www.rayyan.ai; accessed on 31 March 2026) by the first author (MA-R). Duplicate records were identified and removed before screening. The study selection process was conducted independently by two reviewers (MA-R and JS-M) in two stages. First, titles and abstracts were screened according to the predefined eligibility criteria. Second, the full texts of potentially eligible studies were assessed using the PICOS framework. Any disagreements between reviewers were resolved through discussion, and, when necessary, a third reviewer was consulted.

**Figure 1:**
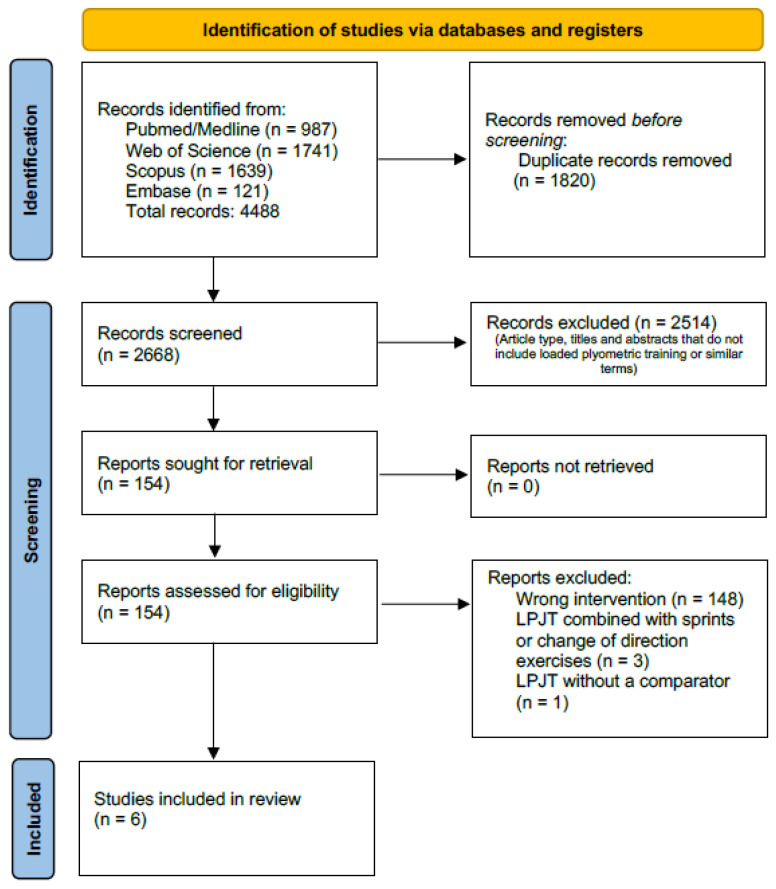
Prisma flow chart of the study selection process.

Data extraction was performed by the first author (MA-R) using a standardized data extraction form and was subsequently checked by a second reviewer (IC-M) to ensure accuracy and consistency. The extracted information included author names, year of publication, study design, participant characteristics (sample size, sex, mean age, competitive level, phase of the season, concurrent soccer-training exposure, and previous plyometric or resistance-training experience), when reported, and details of the plyometric intervention and comparison condition, including exercise type, program duration, training frequency, total intervention period, external load used, and outcomes assessed. When essential data, such as pre- and post-intervention values, were missing or unclear, the corresponding authors were contacted by email to request additional information. If the required data were not obtained, the study was not considered for the corresponding narrative outcome synthesis. The database search yielded a total of 4488 records, of which 1820 were removed as duplicates. A total of 2668 records were screened by title and abstract, and 2514 were excluded. The remaining 154 reports were sought for retrieval and assessed for eligibility in full text. Of these, 148 were excluded: 144 for wrong intervention, 3 because LPJT was combined with sprint training or COD exercises [15,24,25], and 1 because it used a pre-experimental design [16]. A total of six studies were included in the final review (Figure 1).

### 2.5. Risk of Bias and Methodological Quality Assessment

The risk of bias of the included randomized controlled trials was assessed using the revised Cochrane Risk of Bias tool for randomized trials (RoB 2) [26]. Targeting the effect of assignment to intervention. The assessment considered the results contributing to the prespecified physical-performance outcome domains at the immediate post-intervention time point. These domains included horizontal jump performance, vertical jump performance, linear sprint performance, change-of-direction and agility performance, strength- and power-related outcomes, and soccer-specific outcomes, when reported by each trial. RoB 2 evaluates five domains: bias arising from the randomization process, bias due to deviations from intended interventions, bias due to missing outcome data, bias in the measurement of the outcome, and bias in the selection of the reported result. Each domain and the overall risk of bias were classified as “low risk of bias”, “some concerns”, or “high risk of bias”. Although the assessments were conducted with reference to the relevant outcome-specific results, the responses to the signalling questions and the resulting domain-level judgements were identical across the assessed outcomes within each trial. Therefore, the assessments were consolidated into one row per study in the main figure to avoid unnecessary duplication. The outcomes assessed and the study and domain specific supporting rationales are provided in Supplementary Table S2.

Methodological quality was additionally assessed using the Physiotherapy Evidence Database (PEDro) scale, which is commonly used in exercise and rehabilitation intervention studies. The PEDro scale includes 11 items related to eligibility criteria, random allocation, allocation concealment, baseline comparability, blinding procedures, follow-up, intention-to-treat analysis, between-group comparisons, and reporting of point estimates and variability. The first item is not included in the total score; therefore, the final score ranges from 0 to 10. Based on previously established criteria [27,28], studies were classified as having excellent methodological quality when scoring 9–10, good quality when scoring 6–8, fair quality when scoring 4–5, and poor quality when scoring below 4.

The PEDro scale and RoB 2 were used for distinct and complementary purposes. PEDro was retained as a secondary descriptive assessment of selected methodological and reporting characteristics at the trial level, whereas RoB 2 constituted the primary assessment of internal validity and was applied to the outcome-specific results. PEDro scores were not used to exclude studies, weight their findings, determine the risk of bias of individual results, or inform downgrading within the GRADE assessment. The interpretation of the evidence, including the risk-of-bias domain of GRADE and the formulation of the review conclusions, was based primarily on the RoB 2 assessments. Therefore, PEDro and RoB 2 were interpreted as complementary but non-interchangeable instruments.

Two reviewers (MA-R and EG-M) independently assessed risk of bias and methodological quality. Disagreements were resolved through discussion and consensus. When consensus could not be reached, a third reviewer was consulted: LG-L for the RoB 2 assessments and JS-M for the PEDro assessments.

### 2.6. Certainty of Evidence Assessment

The certainty of evidence was assessed independently by two reviewers (MA-R and JS-M) using the Grading of Recommendations Assessment, Development and Evaluation (GRADE) approach [29]. The assessment was performed at the outcome level for the main outcomes included in the narrative synthesis: horizontal jump performance, vertical jump performance, linear sprint performance, COD and agility performance, strength- and power-related outcomes, and soccer-specific outcomes. The results were summarized in a GRADE evidence profile generated using GRADEpro software version 5.0 (https://gradepro.org; accessed on 13 July 2026)). Because the included studies were randomized controlled trials, the initial certainty of evidence was rated as high and was subsequently downgraded, when appropriate, according to the following GRADE domains: risk of bias, inconsistency, indirectness, imprecision, and publication bias. The certainty of evidence was categorized as high, moderate, low, or very low. Separate GRADE profiles were developed for LPJT versus UPJT and LPJT versus a non-training control. Each profile reports the contributing studies and participants, the downgrading decisions, and their rationale.

Certainty was downgraded for risk of bias when a relevant proportion of the evidence for a given outcome came from studies judged as having a high overall risk of bias according to RoB 2. Inconsistency was considered when the direction and magnitude of effects varied across studies or outcome measures, particularly when findings favored different comparison groups or showed trivial or unclear changes. Indirectness was considered when relevant differences were identified in the study population, intervention characteristics, comparison condition, or outcome measures, or when heterogeneous measures were combined within the same outcome domain. Imprecision was considered when the evidence for a given outcome was based on a small number of studies or participants, or when the available comparative estimates were insufficiently precise to support confident interpretation. The absence of pooled estimates was considered only as contextual information and was not used as a standalone criterion for downgrading. Publication bias was considered qualitatively; however, formal assessment using funnel plots or statistical tests was not performed because no meta-analysis was conducted and the number of studies contributing to each outcome was small.

### 2.7. Data Synthesis

Given the limited number of eligible studies and the substantial clinical and methodological heterogeneity across interventions and outcomes, a meta-analysis was not performed. Before deciding on the final synthesis approach, the feasibility of an exploratory quantitative synthesis was examined for outcomes reported by at least two studies. However, quantitative pooling was considered inappropriate because studies differed substantially in participant age and maturation status, competitive level, intervention duration, weekly training frequency, exercise selection, loading magnitude, loading modality, comparator conditions, and outcome-measurement procedures. Only a small number of studies contributed data to the same outcome domain and the same LPJT-versus-comparator contrast.

Outcomes such as standing long jump, countermovement jump, and sprint performance were considered as potential candidates for exploratory pooling or standardized mean-difference calculation. However, apparently similar outcomes were operationalized using different tests, units, or constructs. Sprint performance was reported as sprint velocity in one study and sprint time in others; jump outcomes included squat jump, countermovement jump, vertical jump height, standing long jump, horizontal countermovement-jump variants, and repeated-jump variables; and change-of-direction and agility outcomes were assessed using different tests. Comparator conditions also varied, including LPJT versus UPJT, LPJT versus usual soccer training, and LPJT versus a non-training control. One study compared high- and low-load LPJT conditions with a shared non-training control rather than with UPJT. Pooling these data would therefore have required combining clinically and methodologically non-equivalent comparisons, likely producing summary estimates with limited interpretability.

Accordingly, a narrative synthesis was conducted. Outcomes were grouped into predefined domains: horizontal jump performance, vertical jump performance, linear sprint performance, COD and agility performance, strength- and power-related outcomes, and soccer-specific outcomes. When a study reported multiple measures within the same domain, all relevant measures were described, but the study was considered as a single source of evidence in the overall domain-level interpretation. Reported between-group comparisons were prioritized whenever available. Within-group percentage changes and effect-size estimates were retained only as supplementary descriptive information and were not interpreted as evidence of intervention superiority.

Because the original studies did not consistently report between-group differences in change, associated variance estimates, or corresponding 95% confidence intervals, valid comparative effect estimates could not be calculated consistently across all outcomes. The direction of performance change was interpreted according to the metric reported. For sprint outcomes, a decrease in sprint time and an increase in sprint velocity were interpreted as improvements, whereas an increase in sprint time or a decrease in sprint velocity indicated poorer performance. For other outcomes, the direction of improvement was determined according to whether higher or lower values represented better performance in the original test.

Percentage changes, effect-size estimates, and 95% confidence intervals were extracted when reported in the original studies. Effect sizes were not recalculated, and no additional variance assumptions were applied. Because studies used different statistical approaches and effect-size metrics, these values were used descriptively and were not pooled or interpreted as directly comparable estimates of between-group effects. Given the high overall risk of bias, the small number of studies, and the variability in interventions and outcomes, the findings were interpreted cautiously.

## 3. Results

### 3.1. Study Characteristics

Table 1 summarizes the main characteristics of the included studies. The six included studies were randomized controlled trials [17,18,30,31,32,33] comprising a total of 214 soccer players. Participants were categorized into three age groups: children under 15 years of age (*n* = 92) [18,30], adolescents aged 15–17 years (*n* = 56) [17,32], and adults (*n* = 66) [31,33]. All participants were male; therefore, generalizability to female players remains uncertain. The shared control group in Cao et al. [31] was counted once in the total sample. The mean age of participants ranged from 12.1 ± 2.1 years [30] to 23.41 ± 3.2 years [33].

The competitive level varied across studies and included university soccer players [31,33], elite youth soccer players from a professional club academy [17], U-17 regional league soccer players [32], and young soccer players participating in soccer schools or youth soccer settings [18,30]. Intervention duration ranged from six weeks [17,30,32] to eight weeks [18,31,33]. Training frequency was two sessions per week in five studies [17,18,30,32,33] and three sessions per week in one study [31].

All studies prescribed plyometric training load relative to participants’ BW. Sirin [33], used 1% BW, Kobal et al. [17] and Negra et al. [18] used 8% BW, Niknam et al. [32] applied 10% BW, Cao et al. [31] compared low-load and high-load conditions corresponding to 10% and 20% BW, respectively, and Rosas et al. [30] used individualized handheld weights ranging from 0% to approximately 15% BW.

### 3.2. Risk of Bias and Methodological Quality

**Figure 2:**
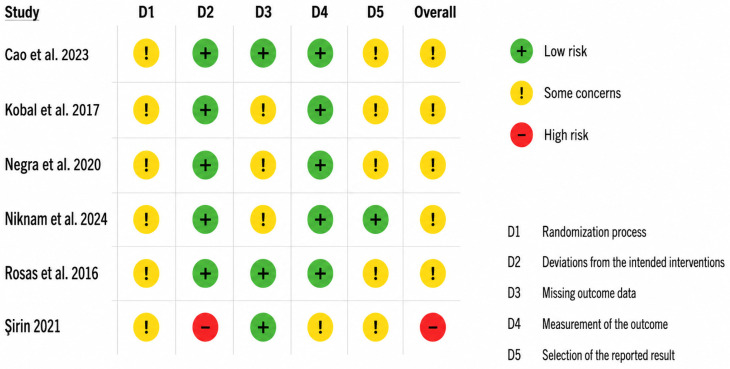
Consolidated risk-of-bias assessments of the included trials using the RoB 2 tool [17,18,30,31,32,33].

Outcome-specific RoB 2 assessments produced identical domain-level judgments across the outcomes evaluated within each trial; therefore, one consolidated assessment per study is presented in Figure 2. Detailed study- and domain-specific supporting rationales are provided in Supplementary Table S2. Overall, five studies were judged as having some concerns, whereas one study was judged as having a high overall risk of bias [17,18,30,31,32,33]. In the randomization-process domain (D1), all six studies raised some concerns. For deviations from intended interventions (D2), five studies were judged as having a low risk of bias, whereas one study was judged as having a high risk of bias. For missing outcome data (D3), three studies were judged as having a low risk of bias and three raised some concerns. Regarding measurement of the outcome (D4), five studies were judged as having a low risk of bias and one raised some concerns. For selection of the reported result (D5), five studies raised some concerns, whereas one study was judged as having a low risk of bias. Regarding methodological quality, assessed using the PEDro scale, four studies demonstrated good methodological quality, with scores of 6/10 [18,30,31,33], whereas two studies demonstrated fair methodological quality, with scores of 5/10 [17,32] (Table 2). PEDro scores were interpreted descriptively and did not modify the outcome specific RoB 2 judgments or the GRADE assessment.

### 3.3. Jumping Ability

Jumping ability was assessed in five studies using horizontal and vertical jump tests, including the standing long jump (SLJ), countermovement jump (CMJ), squat jump (SJ), horizontal CMJ variants, drop jump, and repeated jump outcomes. For horizontal jump performance, LPJT generally produced favorable changes. However, the magnitude of these changes was often comparable to that observed after UPJT. Negra et al. [18] reported similar improvements in SLJ after LPJT and UPJT, with percentage changes of 5.88% and 6.25%, respectively, and identical ES values of 0.50. In contrast, Niknam et al. [32] reported greater improvements in SLJ after LPJT (6.62%; ES = 0.97) than after UPJT (4.26%; ES = 0.73) or control group (2.62%; ES = 0.47). Rosas et al. [30] also reported larger ES values after LPJT than UPJT for right-leg, left-leg, and bilateral horizontal CMJ with arms, with ES values ranging from 0.37 to 0.47, whereas the UPJT group showed ES values ranging from 0.28 to 0.32.

For vertical jump performance, Kobal et al. [17] reported improvements in SJ and CMJ after LPJT, with ES values of 0.78 and 0.78, respectively, compared with 0.50 and 0.50 after UPJT. Niknam et al. [32] also reported greater improvements in vertical jump height after LPJT (12.36%; ES = 1.24) than after UPJT (6.79%; ES = 0.68) or control conditions (2.28%; ES = 0.28). Negra et al. [18] reported larger improvements in CMJ after LPJT (21.52%; ES = 1.00) than after UPJT (12.24%; ES = 0.57). Şirin [33] reported small changes in SJ following LPJT (1.82%; ES = 0.10). Rosas et al. [30] reported improvements in vertical CMJ with arms (7.2%; ES = 0.26) and 20 cm drop jump reactive strength index (19.0%; ES = 0.37) after LPJT, while similar or smaller improvements were observed after UPJT for vertical CMJ with arms (4.3%; ES = 0.26) and 20 cm drop jump reactive strength index (19.0%; ES = 0.20).

Repeated jump outcomes were assessed in one study. Niknam et al. [32] evaluated repeated jump performance using the 15 s repeated jump test and reported improvements after LPJT in the number of jumps (27.85%; ES = 2.54), anaerobic power (35.06%; ES = 2.28), and flight time (30.77%; ES = 2.99). Improvements were also observed in the UPJT and control groups for some repeated jump variables; however, the magnitude and direction of change differed across variables and groups.

### 3.4. Linear Sprint Performance

Linear sprint performance was assessed in three studies using distances ranging from 5 m to 30 m. The comparative findings did not consistently demonstrate an additional benefit of LPJT over UPJT. Kobal et al. [17] assessed sprint velocity over 5 m, 10 m, and 20 m. Because lower sprint velocity indicates poorer performance, the reported decreases reflect performance impairments rather than improvements. Sprint velocity decreased after LPJT, with percentage changes of −5.07%, −3.48%, and −2.54%, respectively, and ES values of 0.92, 0.90, and 0.73. Decreases in sprint velocity were also observed in the UPJT group, with percentage changes of −2.09%, −1.94%, and −1.74%, respectively, and ES values of 0.48, 0.53, and 0.46. Therefore, the positive ES values in this study should be interpreted as the magnitude of the standardized change, not as evidence of improved sprint performance. Negra et al. [18] reported improvements in sprint time after both interventions. The LPJT group showed changes of −7.69%, −9.09%, and −5.26% for the 5 m, 10 m, and 20 m sprint tests, respectively, with ES values of 1.00, 2.00, and 1.00. The UPJT group showed changes of −8.33%, −4.76%, and −2.70%, respectively, with ES values of 1.00, 0.63, and 0.33. Thus, LPJT showed larger effects than UPJT for 10 m and 20 m sprint time, whereas both groups showed similar effects for 5 m sprint time. Şirin [33] reported small changes after LPJT in 10 m and 20 m sprint time (−1.72% and −1.53%; ES = 0.17 and 0.15, respectively), whereas 30 m sprint time increased slightly (0.72%; ES = −0.07). Comparable small changes were observed in the UPJT and control groups. These findings indicate variability in the direction and magnitude of changes across studies and sprint distances, with no consistent pattern favoring LPJT over UPJT.

### 3.5. Change-of-Direction and Agility

COD and agility performance were assessed in two studies. The comparative findings were mixed and did not consistently demonstrate a clear additional benefit of LPJT over UPJT. Negra et al. [18] evaluated COD performance using the Illinois COD test and the modified 505 COD test. In the Illinois COD test, both LPJT and UPJT showed identical percentage changes (−2.15%), although the ES was slightly larger after LPJT than UPJT (0.61 vs. 0.36). In the modified 505 COD test, LPJT showed a larger improvement than UPJT (−6.90%; ES = 2.83 vs. −3.45%; ES = 0.50), suggesting a possible additional benefit of LPJT for this specific COD outcome. Şirin [33] assessed agility performance and reported an improvement after LPJT (−4.75%; ES = 0.92); however, a comparable improvement was also observed after UPJT (−4.10%; ES = 1.10), whereas the control group showed only a small change (−0.42%; ES = 0.09). Taken together, LPJT appeared more favorable than UPJT for the modified 505 change-of-direction test in one study, but the scant evidence does not consistently support the superiority of LPJT for COD or agility performance.

### 3.6. Strength and Power-Related Outcomes

Strength- and power-related outcomes were reported in two studies using different assessment methods. The evidence was limited and did not allow a consistent comparative interpretation of the additional benefit of LPJT over UPJT. Cao et al. [31] assessed angular velocity-related torque variables after high and low load LPJT compared with a passive control condition. The high load LPJT group showed favorable changes in torque peak at 60°/s (11.53%; ES = 0.71) and 180°/s (14.08%; ES = 0.68), as well as reductions in time to reach torque peak at 60°/s (−17.92%; ES = 0.93) and 180°/s (−12.57%; ES = 0.67). The low-load LPJT group also showed favorable changes, particularly in torque peak at 180°/s (26.05%; ES = 0.85). However, because this study did not include an UPJT comparator, these findings cannot determine whether LPJT provides additional benefits over UPJT. Kobal et al. [17] assessed mean propulsive power and reported trivial changes after both LPJT (0.74%; ES = 0.04) and UPJT (1.74%; ES = 0.14). with no indication of a meaningful advantage for LPJT. Taken together, LPJT showed favorable changes in selected torque-related outcomes in one study with a passive control group, whereas comparative evidence against UPJT was limited and did not demonstrate a clear additional benefit for strength- and power-related outcomes.

### 3.7. Soccer-Specific Outcomes

Soccer-specific outcomes were assessed in three studies and included maximal kicking distance, maximal kicking velocity, and dribbling performance. the comparative findings were inconsistent and did not demonstrate a clear additional benefit of LPJT over UPJT for soccer-specific performance. Negra et al. [18] reported larger improvements in maximal kicking distance after LPJT than UPJT (18.00%; ES = 0.90 vs. 11.97%; ES = 0.50). Rosas et al. [30] assessed maximal kicking velocity and observed slightly greater improvements after LPJT (8.30%; ES = 0.34) than after UPJT (6.80%; ES = 0.27) or control conditions (4.00%; ES = 0.20). Şirin [33] evaluated right- and left-foot dribbling performance and reported more favorable changes after UPJT than LPJT. After LPJT, right-foot dribbling time increased by 2.47% (ES = 0.15), indicating poorer performance, whereas left-foot dribbling time decreased by −6.31% (ES = 0.55), indicating improved performance. The UPJT group showed larger improvements in both right-foot dribbling time (−9.44%; ES = 0.85) and left-foot dribbling time (−8.45%; ES = 0.85), whereas the control group showed smaller changes. LPJT appeared more favorable than UPJT for kicking-related outcomes in two studies, but UPJT showed greater improvements in dribbling performance in one study. Therefore, the available evidence does not consistently support the superiority of LPJT for soccer-specific outcomes.

### 3.8. Certainty of Evidence

The certainty of evidence was rated as low for horizontal jump performance and very low for all other outcomes assessed in the GRADE evidence profiles (Supplementary Tables S3 and S4). For horizontal jump performance, certainty was downgraded due to risk of bias concerns and imprecision. For vertical jump performance and linear sprint performance, certainty was downgraded due to risk of bias concerns, inconsistency, and imprecision. For COD and agility performance, certainty was downgraded due to risk of bias concerns, inconsistency, and very serious imprecision. For mean propulsive power in the LPJT versus UPJT comparison, certainty was downgraded due to risk of bias concerns and very serious imprecision. For isokinetic strength outcomes in the LPJT versus non-training-control comparison, certainty was also downgraded due to risk of bias concerns and very serious imprecision. For soccer-specific outcomes, certainty was downgraded due to risk of bias concerns, inconsistency, indirectness, and imprecision. Therefore, certainty was limited by risk of bias concerns, small sample sizes, the limited number of studies contributing to each outcome, imprecision of the available comparative estimates, and inconsistency in the direction and magnitude of effects for several outcomes.

## 4. Discussion

The primary objective of this systematic review was to examine the effects of loaded plyometric-jump training (LPJT) on the physical performance of soccer players. As a secondary objective, this review aimed to compare the effects of LPJT with unloaded plyometric-jump training (UPJT) or passive control conditions. To the best of our knowledge, this is the first review specifically synthesizing evidence on LPJT in soccer players. Six randomized controlled trials were included in the review [17,18,30,31,32,33]. Overall, the available evidence suggests that LPJT may produce favorable changes in selected physical performance outcomes, particularly jumping ability, whereas findings for sprint, COD, agility, strength, power, and soccer-specific outcomes were more variable. However, similar improvements were frequently observed following UPJT, and the magnitude of change varied across studies, outcomes, and testing procedures. Therefore, the current evidence does not allow firm conclusions regarding the superiority of LPJT over UPJT. Comparator conditions differed across studies. Five trials included an UPJT comparator, while three of these trials also included a control group that continued usual soccer training. In contrast, Cao et al. compared two LPJT conditions with a non-training control. Therefore, favorable findings relative to the non-training control should not be interpreted as evidence that LPJT is superior to UPJT or other active training conditions. Moreover, five studies raised some concerns regarding overall risk of bias, whereas one study was judged as having a high overall risk of bias. Methodological quality ranged from fair to good, and the cumulative sample size remained limited (*n* = 214). Accordingly, the findings should be interpreted with caution. The substantial heterogeneity among the included studies should be considered when interpreting the practical applicability of the findings. Participants ranged from children and adolescent soccer players to adults, and the included samples differed in competitive level, including soccer school or youth settings, regional youth players, elite youth academy players, and university soccer players [17,18,30,31,32,33]. These differences may have influenced training responsiveness, because younger players may experience concurrent growth- and maturation-related adaptations, whereas adult players may present more stable neuromuscular profiles and different training histories. In this regard, previous evidence has shown that strength-related capacities and balance performance may differ according to proximity to peak height velocity in young athletes [34]. Biological maturation is an important factor to consider when interpreting the findings of this review, particularly because several included studies involved children and adolescent soccer players [17,18,30,32]. During these developmental periods, improvements in strength, power, sprinting ability, coordination, and neuromuscular control may occur because of normal growth and maturation, independently of the training intervention [35]. Therefore, some of the performance changes observed in younger cohorts may not be attributable solely to LPJT.

Intervention duration also varied from six to eight weeks, and weekly frequency ranged from two to three sessions, which may have affected the magnitude of adaptation across studies. In addition, loading magnitude ranged from very low external loads to approximately 20% of body weight, suggesting that the interventions grouped under the LPJT label may have imposed different mechanical and neuromuscular stimuli. Outcome measures were also heterogeneous, including vertical and horizontal jump tests, sprint time and sprint velocity, COD and agility tests, torque-related variables, and soccer-specific skills. Accordingly, LPJT should not be interpreted as a single uniform intervention. Rather, its effects are likely influenced by participant age, maturation status, competitive level, intervention duration, loading magnitude, loading modality, and outcome specificity. Consequently, the findings of this review should be interpreted as descriptive and hypothesis-generating rather than confirmatory. The current evidence does not establish the superiority of LPJT over UPJT, the optimal external load, a dose–response relationship, or consistent transfer to soccer-specific performance.

### 4.1. Volume, Intensity, and Frequency of LPJT Interventions

The volume, intensity, and frequency of LPJT interventions varied considerably across the included studies. In plyometric training, volume is commonly expressed as the number of ground contacts or jumps per session. Several studies used progressive volume strategies. Kobal et al. [17] increased the number of contacts from 24 to 48 per session, Negra et al. [18] reported volumes ranging from 50 to 120 jumps, Niknam et al. [32] progressed from 80 to 120 jumps, and Rosas et al. [30] reported a range of 96 to 192 jumps per session. In contrast, Cao et al. [31] and Şirin [33] did not report the total number of ground contacts. Rest intervals, supervision, adherence, intervention fidelity, and adverse events were inconsistently reported, limiting assessment of intervention reproducibility and safety. Overall, the reported volumes were generally within ranges commonly recommended for soccer players, including approximately 80 jumps per session with gradual weekly progression [7,22].

External loading intensities ranged from 1% to 20% of BW. Şirin applied a 1% BW load but did not clearly specify the loading method, such as handheld weights, weight plates with handles, or weighted vests. Kobal et al. [17] and Negra et al. [18] used approximately 8% BW, whereas Niknam et al. [32] applied 10% BW using weighted vests. Rosas et al. [30] used individualized handheld external loads of approximately 15% BW, while Cao et al. [31] compared loads corresponding to 10% and 20% BW. However, the direction and magnitude of the responses differed across outcomes, and neither loading condition was consistently superior. Previous recommendations have suggested loads of approximately 8% BW [23]. However, the current low to very low certainty evidence does not support this or any other loading magnitude as optimal. From a practical perspective, very low loads may preserve jump velocity and movement specificity but provide a relatively small overload stimulus. In contrast, heavier loads may increase force-production demands and potentially stimulate strength-related adaptations, but they may also reduce movement velocity, alter jump mechanics, and modify the stretch-shortening cycle demands of the exercise. This distinction is relevant because the outcomes of the included studies did not show a consistent pattern favoring heavier loading strategies. For example, Cao et al. [31] reported favorable changes in selected torque-related outcomes after both high-load and low-load LPJT, whereas the low-load condition showed larger changes for torque peak at 180°/s. Similarly, studies using moderate loads reported favorable changes in some outcomes, but comparable improvements were often observed after UPJT. Nevertheless, findings suggesting potential benefits with higher loads indicate that future studies should examine dose–response effects more systematically.

Training frequency ranged from two to three sessions per week. Most studies implemented two weekly sessions [17,18,30,32,33], whereas one study used three sessions per week [31]. Intervention duration ranged from six weeks [17,30,32] to eight weeks [18,31,33]. Previous recommendations suggest that at least two sessions per week over approximately seven weeks may be required to induce meaningful neuromuscular adaptations [7]. Therefore, shorter interventions or lower training volumes may partly explain the modest or inconsistent effects observed for some performance outcomes after LPJT.

Jumping ability was one of the most frequently assessed outcomes in the included studies, although findings differed between horizontal and vertical jump tests. Overall, LPJT was generally associated with favorable changes in horizontal jump performance, including standing long jump and horizontal countermovement jump variants. These findings are consistent with external literature showing that plyometric-jump training can improve explosive performance [7], Neuromuscular, muscle–tendon, and biomechanical adaptations have been proposed as possible explanations [9,11,12,13,14,36,37]. These findings provide a plausible rationale for the favorable changes observed in horizontal jump outcomes after LPJT, although they should be extrapolated cautiously to chronic adaptations in soccer players.

The relatively favorable horizontal-jump findings may also be related to task specificity, because several protocols included horizontal, unilateral, or multidirectional exercises. External evidence suggests that horizontally oriented training may preferentially benefit horizontal jumping and related tasks [38]. but this mechanism was not directly tested in the included trials.

In contrast, findings for vertical jump performance, including CMJ and SJ outcomes, were less consistent. Some studies reported greater improvements after LPJT, whereas others observed similar or larger changes after UPJT. This variability may be related to differences in external load, exercise selection, training volume, participant characteristics, and technical execution. Differences in external load, exercise selection, training volume, and participant characteristics may have contributed to the inconsistent findings, although the included studies did not directly examine these explanations. Moreover, previous evidence suggests that handheld loading may enhance jumping performance under specific conditions [23,37]. However, UPJT also improved several jump outcomes in the included studies, indicating that unloaded plyometric training may provide a sufficient stimulus when appropriately progressed.

Linear sprint performance showed variable responses across the included studies, with improvements reported after both LPJT and UPJT in some studies, whereas Kobal et al. [17] reported decreases in sprint velocity after both interventions. Therefore, the available evidence does not indicate a clear superiority of LPJT over UPJT for improving sprint performance over distances ranging from 5 to 30 m.

The findings of Kobal et al. [17] further support this cautious interpretation, as sprint velocity over 5 m, 10 m, and 20 m decreased after both LPJT and UPJT, despite improvements in jump performance. In contrast to Kobal et al. [17], Negra et al. [18] reported improvements in linear sprint performance after both LPJT and UPJT, with larger effects after LPJT for 10 m and 20 m sprint performance, whereas the 5 m sprint showed similar effects between groups. These findings indicate that improvements in jump performance did not consistently transfer to sprint performance. External literature proposes rapid force production, stretch-shortening-cycle function, and task-specific force application as possible determinants of sprint adaptation [9,11,39]. These mechanisms were not measured in the included studies, and the additional contribution of external loading remains uncertain.

Variability in loading strategies and exercise selection may partly explain the inconsistent sprint findings. Some included studies used loads of approximately 8–10% of body weight, whereas others applied lower or individualized loads, and the loading modality also differed across interventions [17,18,30,31,32,33]. Although external load may increase force production demands, excessive or poorly selected loading may reduce movement velocity, alter jump mechanics, and compromise the reactive characteristics of plyometric exercises. This is particularly relevant for sprinting, where acceleration performance depends not only on the magnitude of force produced, but also on the ability to apply force effectively in the horizontal direction [40]. Biomechanical evidence from Gold et al. [36] indicates that externally loaded plyometric exercises can modify force-related variables during jumping tasks, while evidence on force-vector specificity suggests that horizontally oriented resistance and plyometric training may be particularly relevant for short-distance sprinting and COD speed [38]. Similarly, Loturco et al. [41] showed that horizontal and vertical plyometric training may produce different sprint transfer effects in high-level U-20 soccer players. Accordingly, the mixed sprint findings observed in the present review may reflect differences in how closely LPJT exercises replicated sprint-specific force vectors and movement velocities. This may help explain why improvements were observed after LPJT in some studies, while similar or greater changes were also reported after UPJT.

COD and agility performance were assessed in a limited number of studies, and the findings did not show a clear advantage of LPJT over UPJT. Improvements were observed after LPJT in some tests; however, similar or greater changes were also reported after UPJT. This suggests that adding external load to plyometric jump training may not necessarily provide a superior stimulus for COD or agility performance when compared with appropriately progressed unloaded plyometric training.

The mixed findings may be explained by the multifactorial nature of COD and agility performance. Previous systematic review evidence indicates that plyometric training can improve agility performance in male soccer players [6]. However, COD speed and agility should not be interpreted as identical constructs. COD speed depends on the ability to decelerate, absorb eccentric forces, re-accelerate, apply force in the appropriate direction, and execute efficient cutting mechanics. Agility, in contrast, also involves perceptual and decision-making components. This distinction has been emphasized by Young et al. [42], who suggested that agility and COD speed should be considered related but distinct abilities. In soccer players, Chaouachi et al. [43] also showed that COD ability is influenced by several physical and technical determinants, which may help explain why improvements in jump or sprint performance do not always translate directly to COD or agility tests.

From a training-specificity perspective, the transfer from LPJT to COD and agility outcomes may depend on whether the exercises reproduce the braking, lateral, horizontal, and re-acceleration demands of the test. Although external loading may increase force production demands, it may also reduce movement velocity or alter technical execution when poorly selected. This is particularly relevant for agility and COD tasks, where rapid force application must be combined with efficient movement control. Consistent with this, Junge et al. [38] highlighted the importance of force-vector specificity, suggesting that horizontally oriented resistance and plyometric training may be especially relevant for short-distance sprinting and COD speed. Therefore, differences in exercise orientation, loading magnitude, and the inclusion of multidirectional or COD-specific stimuli may have contributed to the variability in findings across studies.

Strength- and power-related outcomes were assessed using different measures, including isokinetic torque, rate of force development, and mean propulsive power. Generally, LPJT produced favorable changes in some neuromuscular variables, particularly strength- and torque-related outcomes, although results were inconsistent and often like those observed after UPJT. The potential benefits of LPJT may be explained by the greater mechanical demands imposed by external loading, which can increase force production during the stretch-shortening cycle. Previous evidence indicates that plyometric training enhances neuromuscular function and muscle power [9,10], while loaded plyometric exercises may further increase force-related demands [36]. In addition, power and rate of force development depend on the ability to rapidly generate force [39,44]. However, these findings should be interpreted cautiously because the included studies assessed different neuromuscular constructs. Furthermore, higher external loads may increase force demands but reduce movement velocity, which may explain why LPJT showed benefits in some strength-related outcomes but not consistently in power-related measures compared with UPJT.

Soccer-specific outcomes, including maximal kicking distance, maximal kicking velocity, and dribbling performance, were assessed in a limited number of studies. LPJT was associated with favorable changes in maximal kicking distance and maximal kicking velocity in some studies; however, findings were not consistent across all soccer-specific outcomes. For example, Negra et al. [18] reported larger improvements in maximal kicking distance after LPJT than UPJT, whereas Rosas et al. [30] reported slightly greater improvements in maximal kicking velocity after LPJT than UPJT. In contrast, Şirin [33] reported greater improvements in dribbling performance after UPJT than LPJT. Therefore, the available evidence does not indicate a clear superiority of LPJT for soccer-specific performance.

These findings may be explained by the complex nature of soccer-specific skills, which depend not only on physical capacities but also on technical execution, coordination, and ball-control proficiency. Previous evidence suggests that plyometric training may improve kicking performance in soccer players, probably through improvements in explosive strength and neuromuscular function [8]. However, the transfer from LPJT to kicking or dribbling performance may be limited if the training stimulus does not closely reproduce the technical and coordinative demands of these actions. Thus, the effects of LPJT on soccer-specific outcomes should be interpreted as indirect and task-dependent adaptations.

### 4.2. Potential Limitations and Strengths

This systematic review has several limitations that should be considered when interpreting the findings. According to the outcome-specific RoB 2 assessments, five studies raised some concerns overall, whereas one study was judged as having a high overall risk of bias. The most frequent concerns were related to insufficient reporting of the randomization process, missing outcome data in some trials, and the inability to verify whether the reported results had been selected from prespecified analyses. One study was judged as having a high overall risk of bias because the reported analysis did not adequately preserve the randomized between-group comparison. The fair-to-good PEDro scores should not be interpreted as indicating a low risk of bias for the estimated intervention effects. PEDro summarizes the presence of selected methodological and reporting characteristics at the trial level, whereas RoB 2 evaluates the potential for bias in specific results. Thus, the apparently different classifications are not contradictory and reflect the distinct purposes and structures of the two instruments. GRADE certainty was low for horizontal jump performance and very low for all remaining outcome domains, indicating substantial uncertainty in the comparative effects. Finally, data extraction was checked by a second reviewer but was not performed independently in duplicate, which should also be considered as a methodological limitation.

The small number of included studies and the limited cumulative sample size (*n* = 214) further constrain the robustness and generalizability of the findings. In addition, only a few studies contributed evidence to each outcome domain, limiting firm conclusions regarding the comparative effects of LPJT versus UPJT or passive control conditions. Clinical and methodological heterogeneity should also be considered. The included studies involved soccer players ranging from children to adults, and differences in maturation status, neuromuscular development, and competitive level may have influenced training adaptations. Moreover, LPJT protocols varied in loading magnitude, loading modality, training volume, frequency, duration, and exercise selection, which may partly explain the variability in responses and precluded a quantitative synthesis. Another limitation is the scarcity of evidence on relevant soccer-specific and multidirectional performance outcomes, including postural balance, curved sprinting, repeated-sprint ability, and ecologically valid change-of-direction or agility tasks.

Despite these limitations, this review has several strengths. To the best of the authors’ knowledge, it is the first systematic synthesis specifically examining the effects of LPJT on physical performance in soccer players. A comprehensive search strategy was applied across multiple databases and languages, and established methodological frameworks were used for study selection, outcome-specific risk-of-bias assessment, methodological quality appraisal, and certainty-of-evidence evaluation. In addition, the review provides a structured narrative synthesis across multiple performance domains, allowing a broader interpretation of the potential role of LPJT in soccer conditioning.

## 5. Conclusions

Current evidence on the effects of LPJT on physical performance in soccer players remains uncertain. Low-certainty evidence suggests that LPJT may be associated with favorable changes in horizontal jump performance, whereas evidence for the remaining outcomes is of very low certainty. However, the available evidence does not clearly demonstrate that LPJT is superior to UPJT or passive control conditions. Under appropriately supervised conditions, LPJT may be considered a possible alternative to UPJT for selected performance outcomes. The available evidence does not support specific recommendations regarding loading magnitude, training frequency, or training volume. Given the residual risk of bias concerns and the low to very low certainty of evidence, these findings should be interpreted as descriptive and hypothesis-generating rather than confirmatory.

Future research should include well-designed randomized controlled trials with homogeneous samples, standardized LPJT protocols, and clearly reported loading strategies. Further studies are needed to clarify the effects of different loading magnitudes, loading modalities, exercise orientations, and training durations on physical performance adaptations. Other emerging performance variables, such as curved sprints and postural balance, should also be investigated. Stratified analyses by age, sex, maturity status, and competitive level may help identify which soccer players are most likely to benefit from LPJT.

## Figures and Tables

**Table 1:** Characteristics of studies included.

			Intervention			Results
Author	Study Design	Participants/Age/Sex/Competition Level	Weekly Frequency	Intervention (Sets, Reps, Number of Contacts and % Load)	Other Training/ Exercises	Principal Comparative Findings
Cao et al. [31] High-load LPJT	RCT	High LPJT group: *n* = 10, age: 20.6 ± 1.2 Control group: *n* = 10, age: 19.4 ± 1.7 Sex: Male. University soccer players	3 sessions per week for 8 weeks	High LPJT group: 20% BW load Control group: No athletic training Sets, reps and number of jumps not reported	NR	**↑** High-load LPJT vs. CON. High-load LPJT showed more favorable changes than control in torque peak at 60°/s and 180°/s and in time to reach torque peak at both angular velocities. However, no UPJT comparator was included, so these findings do not demonstrate superiority in favor of LPJT.
Cao et al. [31] Low-load LPJT	RCT	Low-LPJT group: *n* = 10, age: 20.2 ± 1.4 Control group: *n* = 10, age: 19.4 ± 1.7 Sex: Male. University soccer players	3 sessions per week for 8 weeks	Low-LPJT group: 10% BW load Control group: No athletic training Sets, reps and number of jumps not reported.	NR	**↑** Low-load LPJT vs. CON. Low-load LPJT showed more favorable changes than control in selected torque-related outcomes, particularly torque peak at 180°/s. However, no UPJT comparator was included, so these findings do not demonstrate superiority in favor of LPJT.
Kobal et al. [17]	RCT	LPJT group: *n* = 9 UPJT group: *n* = 11 Mean age of sample: 15.9 ± 1.2 Sex: Male Young soccer players	2 sessions per week for 6 weeks	LPJT group: 8% BW load, progressive volume 2–6 sets and 6 reps per exercise. 24–48 jumps per session. UPJT group: Unloaded jumps, Progressive volume 2–6 sets and 6 reps per exercise. 24–48 jumps per session.	Regular soccer training	Mixed/no clear LPJT advantage. Both LPJT and UPJT improved jump performance, but sprint velocity decreased in both groups. The decrease in sprint velocity was numerically greater after LPJT; therefore, no clear additional benefit of LPJT was observed for sprint performance.
Negra et al. [18]	RCT	LPJT Group: *n*: 13, age: 13.0 ± 0.7 UPJT Group: *n* = 16, age: 13.0 ± 0.5 Sex: Male Prepuberal soccer players	2 sessions per week for 8 weeks	LPJT group: 8% BW load, Progressive volume 4–6 sets 6–10 reps per exercise. 50–120 contacts per session. UPJT group: Progressive volume 4–6 sets 6–10 reps per session. 50–120 contacts per session.	NR	**↑** LPJT. LPJT showed larger changes than UPJT for CMJ, 10–20 m sprint time, modified 505 COD, and maximal kicking distance, whereas similar changes were observed for SLJ, Illinois COD, and 5 m sprint time.
Niknam et al. [32]	RCT	LPJT group: *n* = 12, age: 16.8 ± 0.2 UPJT group: *n* = 12, age: 16.8 ± 0.3 Control group: *n* = 12, age: 16.7 ± 0.3 Sex: Male Young soccer players	2 sessions per week for 6 weeks	LPJT group: 10% BW load, progressive volume 4 sets and 5 reps per exercise. Weeks 1–2: 80 jumps Weeks 3–4: 96 jumps Weeks 5–6: 120 jumps UPJT group: Unloaded jumps, progressive volume 4 sets and 5 reps per exercise. Weeks 1–2: 80 jumps Weeks 3–4: 96 jumps Weeks 5–6: 120 jumps Control group: Habitual soccer and physical training	Habitual soccer and physical training	**↑** LPJT. LPJT showed larger changes than UPJT and control conditions for vertical jump height and standing long jump, whereas repeated-jump responses varied across variables and groups.
Rosas et al. [30]	RCT	LPJT group: *n*: 21, age: 12.1± 2.1 UPJT group: *n*: 21, age: 12.3± 2.3 Control group: *n*: 21, age: 12± 2.2 Sex: Male Prepuberal soccer players	2 sessions per week for 6 weeks	LPJT group: ~15% BW load, 2 sets, 4–8 reps per exercise. 96–192 contacts per session. UPJT group: Unloaded, 2 sets, 4–8 reps per exercise. 96–192 contacts per session. Control group: Only soccer training	Regular soccer training	**↑** LPJT. LPJT showed slightly larger changes than UPJT and control conditions for selected horizontal CMJ variants and maximal kicking velocity, whereas changes in vertical CMJ with arms and 20 cm drop jump RSI were similar or outcome specific.
Şirin [33]	RCT	LPJT group: *n* = 12 UPJT group: *n* = 12 Control group: *n* = 12 Age mean sample: 23.41 ±3.2 Sex: Male University soccer players	2 sessions per week for 8 weeks	LPJT group: 1% BW load Progressive volume 3 sets and 6–12 reps. Total jumps not reported. UPJT group: unloaded progressive volume 3 sets and 6–12 reps Total jumps not reported Control group: Regular soccer training	Standard technical and tactical soccer program.	Mixed/**↑** UPJT. Changes in sprint and agility outcomes were generally small or comparable across LPJT, UPJT, and control groups, whereas UPJT showed more favorable changes than LPJT for right- and left-foot dribbling performance.

Abbreviations: NR = not reported; RCT = randomized controlled trial; LPJT = loaded plyometric-jump training; UPJT = unloaded plyometric-jump training; BW = Body weight; CMJ = Countermovement jump; SLJ = Standing long jump. Note: In Cao et al. [31], the same non-training control group was used for both the high-load and low-load LPJT comparisons and was counted only once in the overall sample size. NR indicates that the information was not reported in the original publication. Sprint velocity (m·s^−1^) was reported by Kobal et al. [17] where higher values indicate improvement; the other studies reported sprint time (s), where lower values indicate improvement. ↑ indicates that the principal comparative finding favored the group or condition named after the symbol. Mixed indicates inconsistent or comparable findings across outcomes or groups.

**Table 2:** PEDro scale for the methodological assessment of the included studies.

	Criteria											
Study	1 *	2	3	4	5	6	7	8	9	10	11	Total
Cao et al. [31]	1	1	0	1	0	0	0	1	1	1	1	6/10
Kobal et al. [17]	1	1	0	1	0	0	0	1	0	1	1	5/10
Negra et al. [18]	1	1	0	1	0	0	0	1	1	1	1	6/10
Niknam et al. [32]	1	1	0	1	0	0	0	1	0	1	1	5/10
Rosas et al. [30]	1	1	0	1	0	0	0	1	1	1	1	6/10
Şirin [33]	1	1	0	1	0	0	0	1	1	1	1	6/10

* Criterion not included in the total score.

## Data Availability

No new data were created or analyzed in this study. Data sharing is not applicable to this article.

## Supplementary Materials

The following supporting information can be downloaded at: https://www.mdpi.com/article/10.3390/jfmk11030280/s1, Table S1: Search strategy; Table S2: Outcome-specific RoB 2 assessments and supporting rationales for the included randomized trials; Table S3: GRADE evidence profile: LPJT versus UPJT; Table S4: GRADE evidence profile: LPJT versus a non-training control.

## Abbreviations

The following abbreviations are used in this manuscript:

PT	Plyometric training
LPJT	Loaded plyometric-jump training
UPJT	Unloaded plyometric-jump training
BW	Body weight
SLJ	Standing long jump
CMJ	Countermovement jump
SJ	Squat jump
