# Supplementary material for: Soccer Players’ Physical Performance After Loaded Plyometric-Jump Training: A Systematic Review
Source: J Funct Morphol Kinesiol. 2026 Jul 20;11(3):280. doi: 10.3390/jfmk11030280 (PMC13398299; doi:10.3390/jfmk11030280)
Supplement: Supplementary file 1 [file jfmk-11-00280-s001.zip › jfmk-4416577-supplementary.pdf]

Supplementary Materials

Table S1. Search strategy.

Database	Search strategy	Results
Pubmed/MEDLINE	((“plyometric training” OR “plyometric jump training” OR “loaded plyometric” OR “loaded plyometric jump training” OR “loaded jump” OR “weighted jump” OR “resisted jump” OR “weighted vest” OR “external load” OR “additional load” OR “vertical jump” OR “stretch-shortening cycle”) AND (“soccer players” OR “football players”))	1342
Scopus	TITLE-ABS-KEY (“plyometric training” OR “plyometric jump training” OR “loaded plyometric” OR “loaded plyometric jump training” OR “loaded jump” OR “weighted jump” OR “resisted jump” OR “weighted vest” OR “external load” OR “additional load” OR “vertical jump” OR “stretch-shortening cycle”) AND TITLE-ABS-KEY (“soccer players” OR “football players”)	1639
Web of Science	TS=(“plyometric training” OR “plyometric jump training” OR “loaded plyometric” OR “loaded plyometric jump training” OR “loaded jump” OR “weighted jump” OR “resisted jump” OR “weighted vest” OR “external load” OR “additional load” OR “vertical jump” OR “stretch-shortening cycle”) AND TS=(“soccer players” OR “football players”)	2742
Embase	(‘soccer player’/exp OR ‘football player’/exp OR “soccer players” OR “football players”) AND (‘plyometrics’/exp OR ‘plyometric exercise’ OR ‘plyometric jump training’ OR ‘plyometric training’ OR ‘plyometrics’ OR ‘plyometry’ OR ‘loaded plyometric’ OR ‘loaded plyometric jump training’ OR ‘loaded jump’ OR ‘weighted jump’ OR ‘resisted jump’ OR ‘weighted vest’ OR ‘external load’ OR ‘additional load’ OR ‘vertical jump’ OR ‘stretch shortening cycle’/exp OR ‘stretch-shortening cycle’)	121

Table S2. Outcome-specific RoB 2 assessments and supporting rationales for the included randomized trials.

Study	Results assessed	RoB 2 domain	Judgment	Supporting rationale
**Cao et al. [31]**	Strength- and power-related outcomes: knee extensor peak torque and time to peak torque at 60°/s and 180°/s. Comparison/time point: high-load LPJT and low-load LPJT versus passive control, immediately post-intervention. Effect estimate: post-intervention between-group comparisons and reported changes from baseline.	D1. Randomization process	**Some concerns**	Participants were reported to have been randomly allocated, and the groups were broadly comparable at baseline. However, the method used to generate the allocation sequence and the procedure used to conceal allocation were not described.
		D2. Deviations from intended interventions	**Low risk**	The intervention groups were clearly differentiated, and no important deviations from the assigned interventions were reported. However, the reporting of the statistical analysis combined group-by-time, within-group, and post-intervention comparisons, making the exact assignment-effect estimate less clear.
		D3. Missing outcome data	**Low risk**	Thirty participants were reportedly randomized, but the number of participants included in each group and in each outcome analysis was not reported consistently. Therefore, complete outcome availability for all randomized participants could not be confirmed.
		D4. Measurement of the outcome	**Low risk**	Peak torque and time-to-peak torque were obtained using standardized isokinetic procedures and instrument-generated values applied similarly across groups. Although assessor blinding was not reported, knowledge of group allocation was unlikely to materially influence these objective measurements.
		D5. Selection of the reported result	**Some concerns**	No prospectively available protocol or statistical analysis plan was identified. Multiple isokinetic outcomes, angular velocities, and possible comparisons were available, and the prespecification of the reported analyses could not be verified.
		**Overall risk of bias**	**Some concerns**	There were concerns regarding the randomization process and selection of the reported result, but no domain was judged to be at high risk.
**Kobal et al. [17]**	Vertical jump performance: squat jump and countermovement jump. Linear sprint performance: 5-, 10-, and 20-m sprint velocity. Strength/power: mean propulsive power in the jump squat. Comparison/time point: LPJT versus UPJT, immediately post-intervention. Effect estimate: between-group standardized difference in pre-to-post change.	D1. Randomization process	**Some concerns**	Players were pair-matched according to baseline 20-m sprint performance, and group allocation was performed by tossing a coin. However, allocation concealment was not reported.
		D2. Deviations from intended interventions	**Low risk**	Both groups continued the same soccer training and received the assigned loaded or unloaded plyometric program. No intervention deviations likely to affect the estimated assignment effect were reported.
		D3. Missing outcome data	**Some concerns**	Three of the 12 participants assigned to LPJT were excluded because of injuries that the authors described as unrelated to the intervention or testing, whereas no participants were excluded from UPJT. Analyses were restricted to completers, with no intention-to-treat or sensitivity analysis.

Study	Results assessed	RoB 2 domain	Judgment	Supporting rationale
		D4. Measurement of the outcome	**Low risk**	Jump height was measured using a contact platform, mean propulsive power using a linear transducer, and sprint velocity using electronic light gates under standardized procedures. Assessor blinding was not reported, but the objective measurement methods made material measurement bias unlikely.
		D5. Selection of the reported result	**Some concerns**	No prospectively available protocol or analysis plan was identified. Multiple outcomes and analytic choices were available, and the prespecification of the selected analyses could not be verified.
		**Overall risk of bias**	**Some concerns**	Concerns were identified in the randomization process, missing outcome data, and selection of the reported result. However, no individual domain was judged to be at high risk, and the available information did not indicate that these concerns collectively warranted a high overall risk-of-bias judgment.
**Negra et al. [18]**	**Vertical and horizontal jump performance:** countermovement-jump height and standing-long-jump distance.<br>**Linear-sprint performance:** 5-, 10-, and 20-m sprint time.<br>**Change-of-direction performance:** Illinois COD and modified 505 COD tests.<br>**Soccer-specific performance:** maximal kicking distance.<br>**Comparison/time point:** LPJT versus UPJT, immediately post-intervention.<br>**Effect estimate:** between-group standardized difference in pre-to-post change.	D1. Randomization process	**Some concerns**	Random assignment was reported, and no important baseline differences were identified. However, the sequence-generation method and allocation-concealment procedure were not described.
		D2. Deviations from intended interventions	**Low risk**	The groups received the intended loaded or unloaded programs alongside comparable soccer training, with high reported training compliance. No important deviations likely to affect the assignment effect were identified.
		D3. Missing outcome data	**Some concerns**	Three participants from the LPJT group withdrew from the training center for personal reasons and were excluded from the analyses. The missingness was differential between groups, and no intention-to-treat or sensitivity analysis was reported.
		D4. Measurement of the outcome	**Low risk**	Sprint and change-of-direction outcomes were recorded using electronic photocells, jump performance using standardized objective procedures, and kicking distance using a predefined field protocol. Reported reliability was high, and the same procedures were used across groups. Assessor blinding was not reported, but substantial measurement bias was unlikely.
		D5. Selection of the reported result	**Some concerns**	No prospectively available protocol or statistical analysis plan was identified. Multiple performance outcomes, sprint distances, COD tests, and possible analytical contrasts were available, so prespecification of the reported results could not be verified.
		**Overall risk of bias**	**Some concerns**	Concerns were identified in the randomization process, missing outcome data, and selection of the reported result. However, no individual domain was judged to be at high risk, and the available information did not indicate that these concerns collectively warranted a high overall risk-of-bias judgment.

Study	Results assessed	RoB 2 domain	Judgment	Supporting rationale
**Niknam et al. [32]**	Vertical and horizontal jump performance: vertical jump height and standing long jump.<br>Repeated-jump performance: number of jumps, anaerobic power, and flight time during the 15-s repeated-jump test.<br>Strength/power: isokinetic knee extensor and flexor absolute/relative peak torque, average peak torque, time to peak torque, and average rate of force development at 60°/s and 120°/s.<br>Comparison/time point: LPJT versus UPJT and passive control, immediately post-intervention.<br>Effect estimate: group-by-time/between-group contrast for change from baseline.	D1. Randomization process	**Some concerns**	A shuffled deck of cards was used to generate the allocation sequence, and baseline characteristics were similar across groups. However, the procedure used to conceal allocation was not reported.
		D2. Deviations from intended interventions	**Low risk**	The assigned interventions and usual soccer training were clearly described and matched across groups apart from the intended plyometric exposure. No important deviations from the assigned interventions were reported.
		D3. Missing outcome data	**Some concerns**	One participant from each group was excluded: because of a cold, an ankle sprain during technical training, or self-reported quadriceps pain at post-test. Although losses were balanced and reasons were reported, the complete-case analysis and the post-test pain exclusion leave some uncertainty about outcome-related missingness.
		D4. Measurement of the outcome	**Low risk**	Participants were instructed not to disclose group allocation to examiners. Isokinetic outcomes were measured with a Biodex dynamometer and jump outcomes using standardized instrumented procedures applied similarly across groups.
		D5. Selection of the reported result	**Low risk**	The trial was registered, and the authors stated that no methodological changes were made after trial commencement. The reported jump and isokinetic outcome domains were consistent with the stated study objectives. However, complete prespecification of all individual variables, angular velocities, and analytical contrasts could not be fully verified from the publication alone.
		**Overall risk of bias**	**Some concerns**	The principal concerns related to allocation concealment and complete-case handling of the small amount of missing data; no domain was judged to be at high risk.
**Rosas et al. [33]**	**Horizontal-jump performance:** right-leg, left-leg, and bilateral horizontal countermovement-jump distance with arm swing.<br>**Vertical/reactive-jump performance:** vertical countermovement-jump height with arm swing and reactive strength index during a 20-cm drop jump.<br>**Soccer-specific performance:** maximal kicking velocity.<br>**Comparison/time point:** LPJT versus UPJT and passive control, immediately post-intervention.	D1. Randomization process	**Some concerns**	Participants were randomly assigned using a computer-based procedure, and baseline characteristics were similar across groups. However, the method used to conceal the allocation sequence before assignment was not reported.
		D2. Deviations from intended interventions	**Low risk**	The loaded, unloaded, and soccer-only conditions were clearly defined, and no important deviations from the assigned interventions were reported.
		D3. Missing outcome data	**Low risk**	The reported group sizes were maintained through the post-intervention assessment, and no material outcome-specific missing data were identified.
		D4. Measurement of the outcome	**Low risk**	Horizontal jumps were measured using a fixed tape measure, vertical and drop-jump outcomes using an electronic contact mat, and kicking velocity using a radar gun. Procedures were standardized and applied consistently across groups. Although assessor blinding was not reported, substantial measurement bias was unlikely.

Study	Results assessed	RoB 2 domain	Judgment	Supporting rationale
	**Effect estimate:** group-by-time analysis and between-group difference in pre-to-post change	D5. Selection of the reported result	**Some concerns**	No prospectively available protocol or statistical analysis plan was identified. Multiple jump variants and a soccer-specific outcome were assessed, and prespecification of the selected analyses could not be verified.
		**Overall risk of bias**	**Some concerns**	Concerns were limited mainly to insufficient reporting of allocation concealment and inability to verify prespecified outcome analyses.
Şirin [30]	**Jump performance:** vertical-jump performance and horizontal-jump distance. **Linear-sprint performance:** 10-, 20-, and 30-m sprint time. **Agility and soccer-specific performance:** Illinois agility-test time and right- and left-foot dribbling performance. **Other physical-performance outcomes:** sit-up performance, Yo-Yo level 2, and sit-and-reach flexibility. **Comparison/time point:** LPJT versus UPJT and passive control, immediately post-intervention. **Effect estimate:** reported pre-to-post comparisons and group-level ANOVA results; a clearly specified direct randomized between-group intervention-effect estimate was not consistently reported.	D1. Randomization process	**Some concerns**	The study described random assignment to three groups, but neither the sequence-generation method nor allocation concealment was reported. Baseline comparability was reported.
		D2. Deviations from intended interventions	**High risk**	The study reported pre- and post-intervention group means and ANOVA/Tukey comparisons, but the intervention effect was interpreted largely from separate pre-to-post changes and significance patterns within groups. A clearly defined between-group contrast of change, with an associated uncertainty estimate, was not reported. Consequently, the analysis may not provide an unbiased estimate of the effect of assignment.
		D3. Missing outcome data	**Low risk**	All 36 allocated participants appeared to be included in the reported post-intervention analyses, with no material missing outcome data described.
		D4. Measurement of the outcome	**Some concerns**	Sprint performance was measured with photocells, vertical jump using a jump meter, and the remaining field tests using standardized protocols. However, assessor blinding was not reported, and outcomes such as horizontal jump, sit-and-reach, dribbling, sit-up, and agility involved manual observation, timing, or scoring. Some influence of knowledge of group assignment therefore could not be excluded.
		D5. Selection of the reported result	**Some concerns**	No prospectively available protocol or statistical analysis plan was identified. The study assessed numerous physical, anthropometric, physiological, and biochemical outcomes, with several possible time-point, group, and post-hoc comparisons. Prespecification of the reported results and analyses could not be verified.
		**Overall risk of bias**	**High risk**	The absence of an appropriate direct between-group analysis for the randomized comparison led to a high-risk judgment, with additional concerns regarding randomization reporting, outcome measurement, and selective reporting.

Table S3. GRADE evidence profile: LPJT versus UPJT.

Author(s): MA-R, JS-M, EG-M, IC-M, RR-C, L-G-L
Question: Should loaded plyometric-jump training compared with unloaded plyometric-jump training be used to improve physical performance in soccer players?
Setting: Soccer players
Bibliography: Kobal et al.; Negra et al.; Niknam et al.; Rosas et al.; Şirin

Certainty assessment							№ of patients		Certainty	Importance
№ of studies	Study design	Risk of bias	Inconsistency	Indirectness	Imprecision	Other considerations	LPJT	UPJT		
**Horizontal jump performance (follow-up: range 6 weeks to 8 weeks; assessed with: SLJ and horizontal CMJ variants)**										
4	randomized trials	serious[a]	not serious	not serious	serious[b]	none	58	61	⊕⊕◯◯ low[a,b]	CRITICAL
**Vertical jump performance (follow-up: range 6 weeks to 8 weeks; assessed with: SJ, CMJ and DJ tests)**										
5	randomized trials	serious[a]	serious[c]	not serious	serious[b]	none	67	72	⊕◯◯◯ Very low[a,b,c]	CRITICAL
**Linear sprints performance (follow-up: range 6 weeks to 8 weeks; assessed with: 5 m, 10 m, 20 m, and 30 m sprint tests)**										
3	randomized trials	serious[a]	serious[c]	not serious	serious[b]	none	34	39	⊕◯◯◯ Very low[a,b,c]	CRITICAL
**Change of direction and agility performance (follow-up: 8 weeks; assessed with: Illinois COD test, 505 COD test, and agility test)**										
2	randomized trials	serious[a]	serious[c]	not serious	very serious[d]	none	25	28	⊕◯◯◯ Very low[a,c,d]	CRITICAL
**Strength- and power-related outcomes (follow-up: mean 6 weeks; assessed with: MPP)**										
1	randomized trials	serious[a]	not serious	not serious	very serious[d]	none	9	11	⊕◯◯◯ Very low[a,d]	IMPORTANT

Certainty assessment							№ of patients		Certainty	Importance
№ of studies	Study design	Risk of bias	Inconsistency	Indirectness	Imprecision	Other considerations	LPJT	UPJT		
**Soccer specific outcomes (follow-up: range 6 weeks to 8 weeks; assessed with: Maximal kicking distance, maximal kicking velocity, and dribbling performance tests)**										
3	randomized trials	serious[a]	serious[c]	serious[e]	serious[b]	none	46	49	⊕◯◯◯ Very low[a,b,c,e]	IMPORTANT

Explanations

a. Downgraded by one level because the studies contributing to the outcome raised some concerns or, in some outcome domains, included one study with a high overall risk of bias. The main concerns involved insufficient reporting of the randomization process, missing outcome data in some trials, and uncertainty regarding selection of the reported results

b. Downgraded by one level because the evidence was based on a small number of studies and participants, with limited precision of the available comparative evidence.

c. Downgraded by one level because the direction and magnitude of effects varied across studies and outcomes, with some findings favouring LPJT, others favouring UPJT, and others showing trivial or unclear changes.

d. Downgraded by two levels because the evidence was derived from only one or two small trials with fewer than 60 participants and without sufficiently precise between-group estimates.

e. Downgraded by one level because this outcome combined heterogeneous soccer-specific measures, including kicking distance, kicking velocity, and dribbling performance, which may represent different technical and physical constructs.

Table S4. GRADE evidence profile: LPJT versus a non-training control.

Author(s): MA-R, JS-M, EG-M, IC-M, RR-C, L-G-L
Question: Should loaded plyometric-jump training compared with a non-training control be used to improve isokinetic strength outcomes in soccer players?
Setting: Soccer players
Bibliography: Cao et al.

Certainty assessment							№ of patients		Certainty	Importance
№ of studies	Study design	Risk of bias	Inconsistency	Indirectness	Imprecision	Other considerations	Loaded plyometric-jump training	Non-training control		
**Knee-extensor peak torque (follow-up: mean 8 weeks; assessed with: Peak torque at 60°·s⁻¹ and 180°·s⁻¹)**										
1	randomized trials	serious[a]	not serious	not serious	very serious[b]	none	20	10	⊕◯◯◯ Very low[a,b]	IMPORTANT
**Time to peak torque (follow-up: mean 8 weeks; assessed with: Time to peak torque at 60°·s⁻¹ and 180°·s⁻¹)**										
1	randomised trials	serious[a]	not serious	not serious	very serious[b]	none	20	10	⊕◯◯◯ Very low[a,b]	IMPORTANT

Explanations

a. Downgraded by one level because the single contributing study raised some concerns regarding the randomization process and selection of the reported result.
b. Downgraded by two levels because the evidence was derived from a single small trial with 30 participants and without a sufficiently precise overall comparative estimate.
